# Effects of *Aronia melanocarpa* on Cardiometabolic Diseases: A Systematic Review of Quasi-Design Studies and Randomized Controlled Trials

**DOI:** 10.1900/RDS.2022.18.76

**Published:** 2022-06-30

**Authors:** Christine B. Christiansen, Fredrik B. Mellbye, Kjeld Hermansen, Per B. Jeppesen, Søren Gregersen

**Affiliations:** 1 Department of Clinical Medicine, Aarhus University, Palle Juul-Jensens Boulevard 165, 8200 Aarhus N, Denmark,; 2 Department of Endocrinology and Internal Medicine, Aarhus University Hospital, Palle Juul-Jensens Boulevard 99, 8200 Aarhus N, Denmark,; 3 Steno Diabetes Center Aarhus, Palle Juul-Jensens Boulevard 165, 8200 Aarhus N, Denmark.

**Keywords:** aronia, photinia, polyphenols, diabetes mellitus type 2, cardiovascular risk factors, cardiovascular diseases, clinical trial

## Abstract

**OBJECTIVES:**

Aronia melanocarpa (Aronia) is a shrub with small berries, chokeberries. Chokeberries are claimed to possess health benefits due to a high content of polyphenols. Aronia is known to be extremely antioxidant; however, evidence for its health benefits is not established. This review gives an overview of the impact of Aronia on cardiometabolic risk factors and diseases.

**METHODS:**

Seventeen studies on cardiometabolic risk factors and diseases were identified through a systematic search on PubMed, Embase, and Cochrane. Inclusion criteria were studies with Aronia as intervention, performed in individuals with cardiometabolic disease or risk factors, e.g., type 2 diabetes (T2D), cardiovascular disease, hypertension, dyslipidaemia, impaired glucose tolerance, overweight, central obesity and smoking. Four of these studies were applicable for a quantitative analysis.

**RESULTS:**

Aronia did not influence body weight, circulating triglycerides, total cholesterol, high-density lipoprotein (HDL) cholesterol, or blood pressure. The quantitative analysis revealed a mean reduction in blood glucose of 0.44 mmol/l (P=0.0001) in the treatment group compared with the control group suggesting that Aronia treatment may have a beneficial impact on blood glucose. In addition, treatment durations of 6 weeks to 3 months tended to decrease low-density lipoprotein (LDL) cholesterol, while shorter treatment durations had no effect on LDL cholesterol. The quantitative analysis did not provide data on long-term effects of Aronia on lipids.

**CONCLUSIONS:**

More long-term high-quality randomized controlled studies are needed to clarify if dietary supplementation with Aronia has beneficial effects on cardiometabolic diseases.

## 1 Introduction

Cardiometabolic diseases, e.g., cardiovascular diseases and diabetes, are world-spanning health issues and the primary cause of death worldwide [1,2]. Modifiable risk factors that contribute to development of cardiometabolic diseases include hypertension, dyslipidaemia, obesity, smoking, and impaired glucose tolerance. A decrease in cardiometabolic diseases and related risk factors not only would improve the human health status, but also relieve pressure on our health care systems [2]. Cardiometabolic diseases and related risk factors give rise to oxidative stress. Evidence suggests that a polyphenols-rich diet may have protective abilities and alleviate cardiometabolic diseases and risk factors, possibly by antioxidant mechanisms or by regulation of intracellular signaling pathways, hormones and enzymes [3,4].

*Aronia melanocarpa* is rich in polyphenols and compared to berries of other plants, its berries have the highest concentrations [5]. Due to the high polyphenol content, *Aronia melanocarpa* may be effective in treatment and prevention of cardiometabolic diseases and related risk factors. Interestingly, no studies have reported adverse effects of *Aronia melanocarpa* consumption.

*Aronia melanocarpa* is also called black chokeberry, which refers to the berry’s color and astringent taste. *Aronia melanocarpa* can be confused with *Aronia prunifolia*, which is a hybrid between *Aronia melanocarpa* and *Aronia arbutifolia*, so-called red chokeberry. These species all belong to the Aronia genus and Rosaceae family [6]. In this review, we have chosen to focus on *Aronia melanocarpa* berries, which will be referred to as Aronia throughout this review.

*Aronia melanocarpa* is a 1-2-meter-high deciduous bush [7]. Beside from being an ornamental bush, it is cultivated for its berries that are a rich source of polyphenols, especially anthocyanins [8].

Anthocyanins are highly antioxidant *in vitro* [4], and Aronia berry extracts are claimed to be able to alleviate various diseases. In fact, Aronia berries have been proposed to possess health benefits since ancient times and have been used by Native Americans for treatment of the common cold [6] and in Russia for treatment of arteriosclerosis and hypertension [9]. During the last decade, Aronia has gained its renaissance and several studies have investigated its effect as a treatment for a variety of diseases. In 2011, Aronia gained the health claim “antioxidant effect” from the European Food Safety Authority (EFSA) [10]. However, it remains to be confirmed whether Aronia and polyphenols can exert antioxidant effects *in vivo* [4].

### 1.1 Nutritional values and polyphenols in Aronia

#### 1.1.1 Macronutrients

Sidor et al. provide an overview of the composition of compounds in Aronia berries [11]. The berries contain between 15.3% and 30.8% dry matter (DM). Per 100 g, the berries contain 3.71 g protein, 0.09-0.17 g fat, 13.73-15.06 g carbohydrates, 0.08-1.03 g soluble fibers and 4.01-5.25 g insoluble fibers [11,12]. Carbohydrates include 61.9 mmol sugar /100 g, including 3.54 g glucose, 2.82 g fructose, 0.41 g sucrose and 4.62 g sorbitol [11,12].

#### 1.1.2 Polyphenols

Polyphenols occur naturally in plants as part of the plants’ defense system [3]. In the human diet, polyphenols are primarily found in fruit, vegetables, tea, and coffee [13]. Polyphenols account for Aronia’s antioxidant effect together with vitamins and carotenoids [5]. Procyanidins, anthocyanins, and phenolic acids make up most of the polyphenols in Aronia together with small amounts of flavonols. The concentration of polyphenols in fresh berries depends on cultivar, climate, and harvest seasonality [12,14]. It should be underscored that no gold standard exists for the analyses of polyphenols. Thus, the different methods applied in the many studies probably contribute to the large variation across studies. Typically, the total concentration of polyphenols is determined chromatographically or spectrophotometrically, and the concentration is often expressed as gallic acid equivalents (GAE) [11]. However, chromatography is preferred over spectrophotometry due to a higher specificity [15]. As Table 1 illustrates, spectrophotometrical assays have found the GAE concentration of polyphenols to be within the range of 603-2340 mg/100 g fresh weight (FW) [14,16-23] and 8008-19700 mg/100 g DM [24]. Chromatographic analyses detected 247-2773 mg/100 g FW [17,23,25,26] and 6351-7849 mg/100 g DM, respectively [8,27].

**Table 1.** Concentrations of various polyphenols in Aronia berries, juice and pomace. Adapted and modified from Sidor et al. with implementation of additional studies

	Berries	Juice	Pomace
Polyphenols, measured spectrophotometrically and calculated as gallic acid equivalents	603-2340 mg GAE /100 g FW [14, 16–23]	281-558 mg GAE /100 g FW [18, 39, 41, 46, 51–53, 55–57, 68]	6310 mg GAE/100 g FW [18]
	8008-19700 mg GAE/100 g DM [24]	300.2–663.9 mg GAE/100 g DM [50]	-
Polyphenols, measured chromatographically	247-2773.41 mg/100 g FW [17, 23, 25, 26]	99.63-1123.74 mg/100 g FW [8, 40, 41, 44, 45, 47, 51]	-
	6351.38-7849 mg/100 g DM [8, 27]	3729.07–6686.69 mg/100 g DM [8, 48]	8044–24447.77 mg/100 g DM [31, 48, 66, 67]
Cyanidin-3-O-galactoside	101.080–636.0 mg/100 g FW [16, 17, 25, 28–30]	1.62-149.84 mg/100 g FW [40–44, 47, 52–56, 58–62]	-
	221-1612 mg/100 g DM [8, 14, 31]	408-1451.55 /100 g DM [31, 48]	1119.70-7961.70 mg/100 g DM [31, 48]
Cyanidin-3-O-arabinoside	94.182-299.4 mg/100 g FW [16, 17, 25, 30]	0.66–50.9 mg/100 g FW [42, 53, 55, 57–60]	-
	460-591 mg/100 g DM [31]	114-554.90/100 g DM [31, 48]	454-3116.02 mg/100 g DM [8, 31, 48]
Cyanidin-3-O-glucoside	3.41–46.2 mg/100 g FW [17, 19, 20, 22, 23, 25, 26, 30, 37]	0.05-12.01 mg/100 g FW [38, 40, 42, 51, 53, 55–57, 63, 64, 69]	23.77-52.0 mg/100 g FW [18, 58]
	77.5-89.9 mg/100 g DM [31]	19.71–39.99 /100 g DM [31, 48]	21.0-220.06 /100 g DM [31, 48, 67]
Cyanidin-O-xyloside	9.92–38.2 mg/100 g FW [17, 25, 30]	0.06-5.2 mg/100 g FW [40, 41, 59, 60, 64]	-
	89.7-95.4 mg/100 g DM [31]	17.32–48.35 mg/100 g DM [31, 48]	89.9-275.41 mg/100 g DM [31, 48]
Procyanidin	663.7–1645.64 mg/100 g FW [20, 32]	224.9-392.62 mg/100 g FW [53, 65]	-
	4646-5181.6 per 100 g DM [8, 31]	1408-2371.07 mg/100 g DM [8, 31, 48]	6201.73–13492 mg/100 g DM [8, 31, 48, 67]
Neochlorogenicacid	37.5–115.7 mg/100 g FW [16, 17, 19, 25, 30]	28–154.31 mg/100 g FW [42, 44, 47, 59, 61, 64, 65]	-
	312-346 mg/100 g DM [31]	490-1048.49 mg/100 g DM [31, 48]	220-1174.35 mg/100 g DM [8, 31, 48]
Chlorogenicacid	61-218 mg/100 g FW [8, 16, 17, 23, 25, 30, 35–37]	10.3-138.9 mg/100 g FW [38, 40, 41, 44, 51–53, 59, 63, 64]	42–50 mg/100g FW [63]
	301-642 mg/100 g DM [8, 14, 31]	414–642.74 mg/100 g DM [8, 31, 48]	33.2–1192.69 mg/100 g DM [8, 31, 48, 67]
Flavonols	21.2-71 mg/100 g FW [25, 35]	16.5–21.3 mg/100 g FW [38]	-	
	273.96 mg/100 g DM [27]	-	57.0–126.8 mg/100 g DM [67]

Anthocyanins in Aronia are primarily cyanidin derivatives with O-linked sugar moieties in a 3’-position. These sugars include the monosaccharides xylose, arabinose, glucose, and galactose. Cyanidin-3-O-galactoside is the far most abundant anthocyanin [8,14,16,17,25,28-31], and cyanidin-3-O-arabinoside is the second most abundant [16,17,25,30,31]. The least abundant cyanidin-derivates are cyandin-3-Oglucoside and –xyloside [17,19,20,22,23,25,30-33]. Aronia berries also contain a small amount of the anthocyanin pelargonidin-3-arabinoside [20]. The largest group of polyphenols in Aronia berries is procyanidins [8,20,31,32]. These procyanidins are made of epicatechin monomers, and they are highly polymerized [34]. Around 80% of the procyanidins have a polymerization degree above 10 [20]. Chlorogenic and neochlorogenic acid are the most common phenolic acids in Aronia. The concentration of chlorogenic acid is in general higher [8,14,16,17,23,25,30,31,35-37] than the concentration of neochlorogenic acid [16,17,19,25,30,31]. The flavonol concentration in Aronia is relatively low compared with the other polyphenols which are primarily quercetin glycosides [25,27,35].

Polyphenols are volatile after harvest, and a large part is lost during processing. Among polyphenols, anthocyanins are most prone to degradation [38]. Anthocyanins degrade even at room temperature and the concentration was reduced by 83% over the course of 6 months of storage [38]. Pasteurization at 90 degrees Celsius also reduced the anthocyanin concentration in Aronia juice remarkably. Procyanidins and phenolic acids in Aronia degrade to a much smaller extent during storage and pasteurization [38]. Comparison of polyphenol content in juice with that of fresh berries also clearly demonstrates that both total polyphenol and anthocyanin concentrations are lower in juice than in whole berries. The polyphenol concentration in juice is reduced by 50% [8,18,39-57], and only around one-fourth of the anthocyanins and procyanidins are retained in the juice [31]. As in the whole berries, cyanidin-3-galactoside is the most abundant anthocyanin [31,42,48,53,55,57-60] followed by cyanidin-3-arabinoside [31,40-44,47,48,52-56,58-62]. Likewise, the least abundant cyanidin-derivates are cyandin-3-O-glucoside and –xyloside [31,42,48,53,55,57,59,60]. Phenolic acids are retained well in the juice, and with careful handling, the concentration is around one-third higher in the juice than in the berries [8,31,38,40-42,44,47,48,51-53,59,61,63-65].

The highest concentration of polyphenols is found in the pomace [18,31,48,66,67]. In comparison with the whole berries, with the prerequisite that they are handled identically, the pomace has similar levels of anthocyanins, lower levels of phenolic acids, and high levels of procyanidin [8,31,48,67]. Cyanidin-3-galactoside and -arabinoside are also the most common anthocyanins in the pomace [8,31,48] that also contains smaller amounts of cyanidin-3-xyloside [31,48] and cyanidin-3-glucoside [18,31,48,58,67]. The pomace also contains the phenolic acids neochlorogenic and chlorogenic acid, which are present at relatively equal concentrations [8,31,48,63,67].

#### 1.1.3 Minerals

Aronia berries contain various minerals (Supplementary material Appendix 1, Table 1). Ten of these minerals are considered essential for human health of which Aronia berries contain relevant concentrations of 3, i.e., calcium, potassium, and magnesium with amounts of 1212, 6790, and 669 mg/ kg DM, respectively [70]. A daily intake of 100 g berries covers 12%-20% of the recommended daily intake of these 3 minerals. The berries also contain small amounts of possible toxic metals, such as heavy metals that the plant absorbs from the soil, but the amount is far below the upper limit suggested by the World Health Organization [70-72].

#### 1.1.4 Vitamins

Vitamins are extremely important in multiple processes in the human body [73]. Aronia primarily provides vitamin C and various forms of vitamin A. Aronia berries contain 4.86 mg carotenoids per 100 g of which 1.67 mg are beta-carotene and 1.22 mg are beta-cryptoxanthin [74]. These compounds are antioxidants and precursors of vitamin A. According to Burri et al., beta-cryptoxanthin is found only in a few fruits and vegetables, but it is more bioavailable than the far more common compound, beta-carotene. In comparison, the concentration of beta-cryptoxanthin in Aronia berries is high; the vegetables with the highest concentrations of beta-cryptoxanthin are different squash species with 1.12 mg to 3.47 mg and persimmons with 1.45 mg per 100 g [75]. On the other hand, many vegetables are richer sources of beta-carotene than Aronia; these vegetables include, for example, sweet potatoes, which contain around 7 mg/100 g [76]. Supplementary material Appendix 1, Table 2 shows the concentrations of vitamins present in Aronia berries [34,77].

**Table 2.** An overview of the human intervention trials identified through the systematic search

Study	Participants	Study duration	Daily Aronia treatment	Study type	Findings
Kardum et al. 2015 [56]	23 individuals with mild hypertension	4 weeks	100 ml Aronia juice corresponding to around 600 mg phenolics per day for 4 weeks	Uncontrolled, quasidesign	↔FBG, ↔TG, ↓TC, ↔LDL, ↔HDL, ↓DBP, ↓SBP
Kardum et al. 2014 [68]	20 women with central obesity	4 weeks	100 ml Aronia juice corresponding to around 600 mg phenolics per day for 4 weeks	Uncontrolled, quasidesign	↔BW, ↔FBG, ↔TG, ↔TC, ↔LDL, ↓HDL, ↔PUFA, ↑3-n PUFA, ↔DBP, ↓SBP
loo et al. 2016 [86]	37 individuals with mild hypertension	8 weeks	Aronia juice or powder corresponding to around 2000 or 300 mg polyphenols, respectively	RCT (crossover)	↔BW, ↔FBG ↔TG, ↔TC, ↔HDL, ↔DBP, ↔SBP, ↔CRP, ↔IL-4, , ↔IL- 5, ↔IL-6, ↔IL-7, ↔IL-8, ↓IL-10, ↔IL- 4, ↔TNF-α,
Broncel et al. 2010 [87]	25 individuals with MS	2 months	300 mg Aronia extract	Uncontrolled, quasidesign	↔BW, ↔FBG, ↓TG, ↓TC, ↓LDL, ↔HDL, ↓DBP, ↓SBP, ↔CRP, ↔FGN
Sikora et al. 2014 [88]	25 individuals with MS	2 months	300 mg Aronia extract	Uncontrolled, quasidesign	↔BW, ↓TC, ↓LDL, ↔HDL, ↔DBP, ↓SBP, ↔CRP, ↓ACE
Sikora et al. 2012 [89]	38 individuals with MS	2 months	300 mg Aronia extract	Uncontrolled, quasidesign	↔BW, ↓TC, ↓LDL, ↔HDL
Xie et al. 2017 [90]	25 former smokers	12 weeks	500 mg Aronia extract	RCT	↔BW, ↔TG, ↔TC, ↓LDL, ↔HDL, ↑DBP, ↔SBP, ↔IL-1β, ↔IL-6, ↔TNF-α, ↔MCP-1
Milutinovic et al. 2019 [91]	35 T2D patients	3 months	150 ml Aronia juice containing around 600 mg polyphenols	Uncontrolled, quasidesign	↓FBG, ↔HbA1c, ↓BW, ↔TG, ↔TC, ↓LDL, ↔HDL, ↔DBP, ↔SBP, ↔CRP
Tasic et al. 2021 [92]	143 subjects	4 weeks	400 mg Aronia extract	Uncontrolled, quasidesign	↓FBG, ↓BW, ↔TG ↓TC, ↓LDL, ↔HDL, ↓DBP, ↓SBP, ↔CRP,
Skoczynska et al. 2007 [95]	58 men with mild hypercholesterolaemia	6 weeks	250 ml Aronia juice	Uncontrolled, quasidesign	↓FBG, ↓TG, ↓TC, ↓LDL, ↔HDL, ↓DBP, ↓SBP, ↔CRP, ↓FGN
Duchnowicz et al. 2018 [96]	77 children or adolescents with MS	2 months	300 mg Aronia extract	Uncontrolled, quasidesign	↓TG, ↓TC, ↓LDL, ↑HDL,
Pokimica et al. 2019 [97]	84 individuals with BMI ≥ 25 kg/m^2^ OR SBP/DBP120/80 OR hypercholesterolaemia	4 weeks	300 mg Aronia extract	RCT	↔BW, ↔TG, ↔TC, ↔LDL, ↔HDL, ↑PUFA, ↑3-n PUFA, ↔oxLDL
Ryszawa et al. 2006 [98]	15 individuals with risk of atherosclerosis			Ex vivo	↓blood coagulation
Narauszewicz et al. 2007 [99]	44 patients with coronary artery disease that are treated with statins	6 weeks	255 mg Aronia extract	RCT	↔BW, ↔FBG, ↔TG, ↔TC, ↔LDL, ↔HDL, ↓DBP, ↓SBP, ↓CRP, ↓IL-6, ↓MCP-1, ↓oxLDL
Duchnowicz et al. 2012 [100]	25 individuals with hypercholesterolaemia	2 months	300 mg Aronia extract	Uncontrolled, quasidesign	Lipid peroxidation
Turnovska et al. 2014 [103]	10 obese individuals	2 months	170-200 ml Aronia juice	Uncontrolled, quasidesign	↓resistin, ↓leptin, ↑adiponectin
Simeonov et al. 2002 [104]	25 T2D patients	3 months	150 ml Aronia juice	RCT	↓FBG, ↓HbA1c, ↓TC, ↓DBP, ↓SBP

Abbreviations: FBG: fasting blood glucose, HbA1c: haemoglobin A1c, TG: triglycerides, TC: total cholesterol, LDL: low density lipoprotein cholesterol, HDL: high density lipoprotein cholesterol, PUFA: polyunsaturated fatty acid, DBP: diastolic blood pressure, SBP: systolic blood pressure, CRP: C-reactive protein, IL: interleukin, TNF-α: tumor necrosis factor-α, MCP-1: monocyte chemoattractant protein-1, oxLDL: oxidized low density lipoprotein, FGN: fibrinogen, ACE: angiotensin-converting enzyme, ↔ : no change, ↑ increase, ↓ decrease

### 1.2 Proposed mechanisms for cardiometabolic effect of Aronia

It has been established that polyphenols are both anti-inflammatory and antioxidant *in vitro* and in animal models; however, regarding human health, studies provide conflicting results, and the mechanisms by which plant polyphenols may alleviate cardiometabolic risk factors and diseases are not fully understood [4]. Under normal, healthy conditions, the body’s redox system is in balance, i.e., the number of antioxidants is sufficient to neutralize the reactive oxygen species and reactive nitrogen species [78]. Evidence suggests that imbalances in the redox system play an important role for the cardiometabolic risk factors and diseases, not only in the disease pathogenesis but also before the disease becomes symptomatic [79,80]. However, whether increased intake of antioxidants alleviates oxidative stress remains unresolved [78]. Even though Aronia polyphenols possess strong antioxidant activity *in vitro*, there is no evidence that substantiates a direct antioxidant effect in humans where Aronia acts as radical scavenger [4]. An indirect antioxidant effect has also been proposed through which polyphenols reduce oxidative stress and prevent inflammation via enhancement of antioxidant enzymes such as superoxide dismutase and glutathione peroxidase as well as inhibition of pro-oxidative enzymes such as xanthine [81]. Also, a comprehensive interplay between polyphenols and various intracellular signaling pathways may reduce inflammation by increasing the expression of anti-inflammatory cytokines such as IL-4, ILl-10 and TGF-β, as well as decreasing the expression of pro-inflammatory cytokines such as IL-1β, IL-2, IL-6 and TNF-α [81, 82].

Mechanisms that more directly improve cardiometabolic health have also been suggested. Thus, evidence suggests that polyphenols can increase nitric oxide concentrations. This both protects the endothelium against arteriosclerosis and lowers the blood pressure [82]. Thus, one study where chlorogenic acid-rich green coffee extract was administered for 12 weeks decreased both systolic and diastolic blood pressure [83]. Furthermore, it has been proposed that polyphenols stimulate peroxisome proliferator-activated receptor-α which in turn stimulates glucose uptake and improve insulin sensitivity [84].

In addition to providing the aforementioned overview of the content and composition of substances in Aronia, our aim is to present a meta-analysis on the impact of Aronia on cardiometabolic metabolism, risk factors, and diseases, with a special focus on the impact of polyphenols.

## 2 Methods

### 2.1 Data sources and study selection

Studies were identified through a systematic search in PubMed, Embase, and Cochrane. The search included terms such as chokeberry, photinia and Aronia that were searched as “all fields” and MeSH terms. The search was performed until January 3, 2022. All studies investigating the effects of Aronia and performed in humans with cardiometabolic diseases, e.g., type 2 diabetes (T2D), cardiovascular disease, hypertension, dyslipidemia, impaired glucose tolerance, overweight, central obesity, and smoking, were included independently of study duration, study design, and Aronia dosing. Outcomes were biomarkers of cardiometabolic diseases including but not limited to blood pressure, blood lipids, and inflammatory markers. However, studies with Aronia administered in a combination treatment with other substances and only constituted a minor amount were not included. Only studies with a control group were included in the quantitative analysis. Studies not performed in humans and studies reported in other languages than English as well as studies performed in humans without cardiometabolic risk or risk factors were excluded.

Two persons (CBC and FBM) reviewed all studies for eligibility – first, based on title and abstract, and second, on the full article. Mean ± SD values for post-treatment were extracted for both intervention and control groups, along with the number of participants in each group. Cholesterol, triglyceride, and glucose concentrations were given in mmol/l and HbA1c in percentages.

Due to a limited number of studies in this area, results from studies that could not be included in the quantitative analysis have been reported applying a narrative approach. These studies comprise quasi-designed studies and cross-over studies.

### 2.2 Quality assessment

The quality of the studies included in the quantitative analysis was assessed by 2 persons (CBC and FBM) using the Cochrane Collaboration’s tool for assessing risk of bias [85]. The risk of selection, performance, detection, attrition, and reporting bias was evaluated and rated as low risk, high risk, or unclear risk.

### 2.3 Statistical analysis

We used RevMan 5.4 for statistical analyses of differences in the post-treatment mean ± SD values between the intervention and the control groups. The random effects model was used to determine the overall effect, which was given as mean difference ± 95 % CI for the studies and illustrated in a forest plot. I^2^ statistics were used to evaluate heterogeneity between studies. I^2^ values of 25%, 50%, and 75% are considered low, moderate, and high heterogeneity, respectively.

## 3 Results

**Figure 1.**
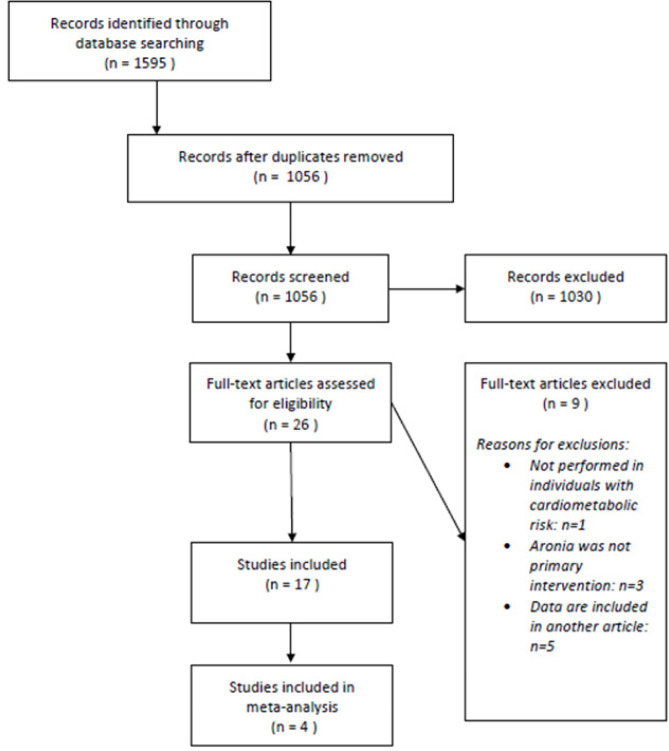
Flow chart of trial search.

**Figure 2.**
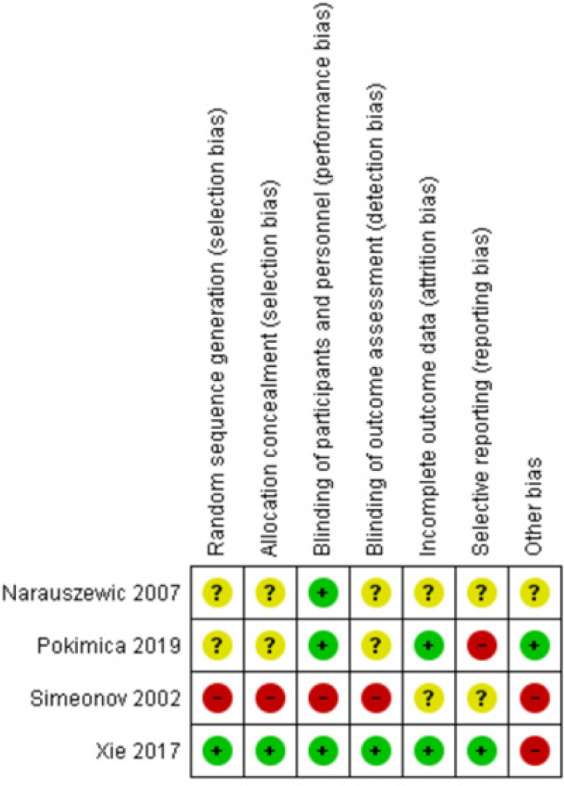
Risk of bias summary.

The systematic search identified 1059 records after removal of duplicates, of which 26 were potentially relevant, as Figure 1 illustrates. Of these, 9 studies were excluded as they were in other language than English, were not performed on humans, did not have Aronia as primary treatment, or were not performed in persons with increased cardiometabolic risk. Seventeen studies were conducted on persons with cardiometabolic diseases which are shown in Table 2. Of these studies, 4 studies could be included in the quantitative analysis. A cross-over study fulfilled all criteria, but due to the design, it cannot be included in the analysis. For the studies included in the quantitative analysis, the risk of bias is summarized in Figure 2. Because only 4 studies could be included in the quantitative analysis, the finding from the remaining 13 studies is also described and an overview is given in Table 3.

**Table 3.** An overview of the effect of Aronia by outcome. For each outcome, the number of studies that found either no change (↔), an increase (↑) or a decrease (↓) is stated along with a reference to the studies

BW	FBG	HbA1c	TG	TC	LDL
7↔ [86-90, 97, 99]3↓ [68, 91, 92]	6↔ [56, 68, 86, 87, 97, 99 4↓ [91, 92, 95, 104]	1↔ [91]1↓ [104]	8↔ [56, 68, 86, 90-92,97, 99]3↓ [87, 95, 96]	6↔ [68, 86, 90, 91,99, 97]8↓ [56, 87-89, 92, 95,96, 104]	4↔ [56, 68, 97, 99]8↓ [87-92, 95, 96]
**HDL**	**DBP**	**SBP**	**CRP**	**IL-6**	**TNF--α**
10↔ [56, 86-92,95, 97, 99, ]1↑ [96]1↓ [68]	4↔ [68, 86, 88, 91]5↓ [56, 87, 92, 95, 104]1↑[87]	3↔ [90, 91, 97]7↓ [56, 68, 87, 88,92, 95, 99]	6↔ [86-88, 91, 92, 95]1↓ [99]	2↔ [86, 90]1↓ [99]	2↔ [86, 90]
**MCP-1**	**oxLDL**	**FGN**			
1↔ [90]1 ↓ [99]	1↔ [97] 1↓ [99]	1↔ [88] 1↓ [99]			

Abbreviations: BW: body weight, FBG: fasting blood glucose, HbA1c: haemoglobin A1c, TG: triglyceride, TC: total cholesterol, LDL: low density lipoprotein cholesterol, HDL: high density lipoprotein cholesterol, PUFA: polyunsaturated fatty acid, DBP: diastolic blood pressure, SBP: systolic blood pressure, CRP: C-reactive protein, IL: interleukin, TNF-α: tumor necrosis factor-α, MCP-1: monocyte chemoattractant protein-1, oxLDL: oxidized low density lipoprotein, FGN: fibrinogen, ACE: angiotensin-converting enzyme.

### 3.1 Systematic review

#### 3.1.1 Aronia’s effect on overweight and obesity

Three quasi-designed studies with a total of 88 obese (baseline BMI 30.9-31.1 kg/m^2^) individuals with metabolic syndrome (MS) and one cross-over study with 37 hypertensive participants did not observe weight loss after treatment with 300 mg to 2000 mg extract/ day for 2 months [86-89]. Neither overweight former smokers nor obese individuals with MS had reductions in waist circumference after treatment with 300-500 mg Aronia extract per day for 2 to 3 months [87,88,90]. However, 3 quasi-designed studies found an effect of Aronia on body weight [68,91,92]. Thirty-five enrolled T2D patients had a small significant reduction in BMI from 28.8 to 28.4 kg/m^2^ upon treatment with Aronia juice corresponding to around 600 mg polyphenols per day for 3 months [91]. Similarly, 20 enrolled women with central obesity and a BMI of 36.1 kg/m^2^ at baseline lost an average of 1.1 kg/m^2^ and had a reduction of 4.2 cm waist circumference after treatment with 100 ml Aronia juice per day, corresponding to around 600 mg polyphenols, for 4 weeks [68] (Table 3).

#### 3.1.2 Aronia’s effect on blood glucose and type 2 diabetes

Impaired fasting blood glucose is defined as fasting glucose values between 6.1 and 6.9 mmol/l, whereas a person is considered diabetic when values exceed 6.9 mmol/l [93]. In women with central obesity and impaired fasting glucose, administration of 100 ml juice, corresponding to 600 mg polyphenols, for 4 weeks did not reduce fasting blood glucose compared to baseline [68]. In contrast, a quasi-designed study found an improvement in glycaemia in persons with MS or T2D after administration of Aronia extract, corresponding to 400 mg polyphenols, for 4 weeks [92]. Furthermore, in 35 persons with T2D, treatment with 150 ml Aronia juice containing around 600 mg polyphenols per day for 3 months in combination with their usual medicine induced a slight, significant reduction in fasting blood glucose from 8.0 mmol/l at baseline to 7.6 mmol/l, while HbA1c was unchanged [91].

Hypoglycaemia, defined as a blood glucose below 3.9 mmol/l, is a feared side effect of diabetes treatment [94]. The impact of Aronia on fasting blood glucose in normoglycaemic persons has been examined in- 3randamized controlled trials and 4-quasi experimental studies, and in all cases fasting blood sugar remained far above 3.9 mmol/l [56,68,85-87,89,97]. Hypercholesterolaemic men treated with 250 ml Aronia juice, corresponding to around 400 mg polyphenols, daily for 6 weeks had a small reduction in fasting blood glucose from 5.5 mmol/l at baseline to 5.1 mmol/l [95]. In the remaining 3 studies, baseline values of fasting blood glucose of 5.0 mmol/l to 5.6 mmol/l were not changed after treatment with Aronia powder or juice in doses corresponding to between 300 mg and 2000 mg polyphenols per day for 4 to 9 weeks [56,68,86].

#### 3.1.3 Aronia’s effect on cardiovascular risk factors and diseases

##### 3.1.3.1. Blood lipids

Three quasi-designed studies performed in individuals with MS or hypercholesterolaemia detected a decrease in triglyceride (TG) concentration after administration of Aronia extract or juice for 6 weeks to 2 months [87,95,96]. One study used 250 ml of juice containing an unknown concentration of polyphenols [95], while the remaining studies used an Aronia berry extract with a daily dose of 300 mg polyphenols [87,96]. In contrast, 4 other quasi-designed studies and one cross-over study performed in individuals with MS, hypertension, or T2D did not detect differences even though the authors administered juice containing from 300mg to 2000 mg polyphenols/day for 4 weeks to 3 months [56,68,86,91,92].

Seven quasi-designed studies performed in individuals with hypercholesterolaemia, MS, hypertension or T2D found that Aronia extract administered in daily doses of 300 mg to 600 mg polyphenols for 4 weeks to 3 months lowered total cholesterol [56,87,89,90,92,95,96]. In contrast, 2 quasi-designed studies and a cross-over study did not detect any effect on total cholesterol after administration of 300 mg to 2000 mg polyphenols /day for 4 to 8 weeks [68,86,91].

Overall reduction in LDL cholesterol after longer-term treatment with Aronia has been reported [87-89,91,92,95,96]. In general, LDL cholesterol levels are reduced by Aronia supplementation in individuals with hypercholesterolaemia, MS, and T2D upon treatment with 300 mg to 600 mg polyphenols per day for 6 weeks to 3 months [87-89,91,92,95,96]. However, individuals with hypertension and obesity treatment with 600 mg polyphenols daily for shorter periods did not elicit a lowering of LDL cholesterol [56,68]. One study performed in persons with MS or T2D found a decrease in LDL cholesterol after 4 weeks’ treatment [92]. Among 9 studies, Aronia was only capable of inducing increased HDL cholesterol in a quasi-designed study performed in individuals with MS after treatment with 300 mg polyphenols for 2 months, while other comparable studies did not detect any positive effect [56,86,87-89,91,92,95,96].

Evidence suggests that Aronia extract can increase plasma polyunsaturated fatty acid (PUFA) concentrations. In individuals with cardiovascular risk, 4 weeks Aronia supplementation resulted in an overall increase in plasma PUFA in a RCT [97]. In contrast, a similar quasi-designed study did not observe any changes in PUFA concentrations [68]. However, the studies detected an increase in 3-n PUFAs which are considered the fraction of PUFA with the highest health impact [68,97].

##### 3.1.3.2. Blood pressure

Aronia has shown positive effects on blood pressure in quasi-experimental designed studies. Out of 7 studies, all but one observed positive effect on diastolic blood pressure (DBP), and in all but 2, in systolic blood pressure (SBP) after supplementation with Aronia corresponding to around 300 mg to 800 mg polyphenols per day administered for 4 weeks to 2 months [56,68,87,88,92,95]. Study populations were composed of individuals with MS [87,88,92], hypercholesterolaemia [95], T2D [91] hypertension [56], or central obesity [68].

##### 3.1.3.3. Blood coagulation

Evidence suggests that Aronia can inhibit coagulation by platelet-mediated actions. Its anticoagulant activity has not been examined directly in humans, but in an *ex vivo* study using platelets from persons with increased cardiovascular risk, Aronia was able to reduce coagulation [98].

#### 3.1.4 Aronia’s effect on markers of inflammation and oxidation

In an RCT, 6 weeks treatment with Aronia significantly reduced C-reactive protein (CRP) from 4.5 to 3.5 mg/l in patients with coronary artery disease [99]. In 2 other RCTs [86,90] and in 4 quasi-designed studies [87,88,92,95] in individuals at cardiovascular risk, e.g., smoking, MS, hypercholesterolaemia, and hypertension, Aronia did not have any effect on inflammatory markers after 6 to 12 weeks of treatment.

RCTs also have investigated Aronia’s effect on other inflammatory markers with discordant results. Loo et al. [86] detected a decrease in IL-10 and TNF-α after 8 weeks of Aronia treatment, but no change in IL-4, -5, -6, -7, -8, or -13 in individuals with hypertension. In patients with coronary artery disease, treatment with Aronia for 6 weeks reduced IL-6 and Monocyte chemoattractant protein-1 (MCP-1) concentrations [99]. However, in former smokers IL-6, IL-1β, MCP-1 and TNF-α concentrations remained unchanged after 12 weeks of Aronia treatment [90].

In individuals with cardiovascular risk, an Aronia supplementation of 300 mg polyphenols per day for 2 months resulted in decreased lipid peroxidation of 40% [100]. In a RCT performed in 44 patients with coronary artery disease treated with statins, 6 weeks treatment with Aronia decreased oxLDL cholesterol [99]. Conversely, in a quasi-experimental study of 84 individuals at increased cardiovascular risk, Aronia did not lower oxLDL cholesterol following a 4-week treatment [97].

#### 3.1.5 Aronia’s effect on other compounds with implications in cardiometabolic health

Increased circulating fibrinogen levels are believed to be correlated to enhanced risk of cardiovascular diseases [101]. In a quasi-designed study, Aronia juice reduced fibrinogen levels in men with hypercholesterolaemia after 6 weeks treatment [95]. Surprisingly, another quasi-designed study in persons with MS detected an increase after 2 months treatment [87]. In addition, dysregulation of adipokines is connected to cardiometabolic diseases. Increased adiponectin lowers the risk of diabetes, and adiponectin levels are decreased in individuals with T2D [102]. In contrast, increased resistin increases the risk of T2D and atherosclerosis. Leptin plays a role in the pathogenesis of both diabetes and obesity [102]. A small pilot study with obese patients suggests that Aronia extract might influence these hormones, as 2-month treatment resulted in a decrease in resistin and leptin as well as an increase in adiponectin [103]. Aronia also may exert cardioprotective effects through angiotensin-converting enzyme (ACE) inhibition. Sikora et al. reported up to 30% reduction in ACE activity in individuals MS after 2 months treatment with Aronia [88].

### 3.2 Quantitative analysis

**Figure 3.**
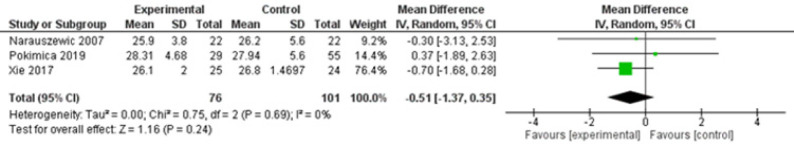
Forrest plot of post-treatment BMI in kg/m^2^ (mean difference ± SD) between the intervention and the control groups. Abbreviations: CI: Confidence interval.

The impact of Aronia on overweight has been studied in 3 RCTs including 190 overweight persons (baseline BMI 25.6-27.4 kg/m^2^) being at cardiovascular risk, e.g., former smoking, previous myocardial infarction, hypertension, or hypercholesterolaemia [90,97,99]. Study duration varied from 4 to 12 weeks during which patients received extracts or juice corresponding to between 300 mg and 2000 mg polyphenols per day. The studies did not detect any weight reducing effect of Aronia, which was confirmed in the meta-analysis as Figure 3 illustrates.

**Figure 4.**
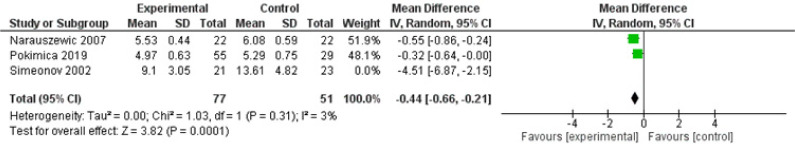
Forrest plot of post-treatment fasting blood glucose in mmol/l (mean difference ± SD) between the intervention and the control groups. Abbreviations: CI: Confidence interval.

With respect to blood glucose, a controlled study with 21 individuals with T2D, observed that treatment with 200 ml Aronia juice for 3 months decreased fasting blood glucose from 13.3 mmol/l to 9.1 mmol/l and HbA1c from 9.4% to 7.5% [104]. Importantly, both fasting blood glucose and HbA1c remained unchanged in persons with T2D not receiving Aronia. The same study also found a reduction in blood glucose 60 minutes after drinking a single dose of 200 ml Aronia juice, indicating that Aronia has an acute effect on blood glucose. On the other hand, after consumption of a standard breakfast together with Aronia, there was no effect on postprandial blood glucose in persons with T2D or Type 1 diabetes [104]. The impact of Aronia on fasting blood glucose in normoglycaemic individuals has been examined in 2 studies with durations of 4 to 6 weeks, but supplementation with Aronia did not decrease fasting blood glucose [97,99]. However, even though there seems to be no overall effect of Aronia on blood glucose, the quantitative analysis found a mean reduction of 0.75 mmol/l [-0.66, 0.21] (P = 0.04), but the level of heterogeneity between the studies was high (I^2^ = 84%). Due to the high risk of bias in one of the studies [104], a new analysis was performed without this study. The new analysis showed that Aronia supplementation reduced the blood glucose 0.44 mmol/l (P = 0.0001) as Figure 4 depicts.

**Figure 5.**
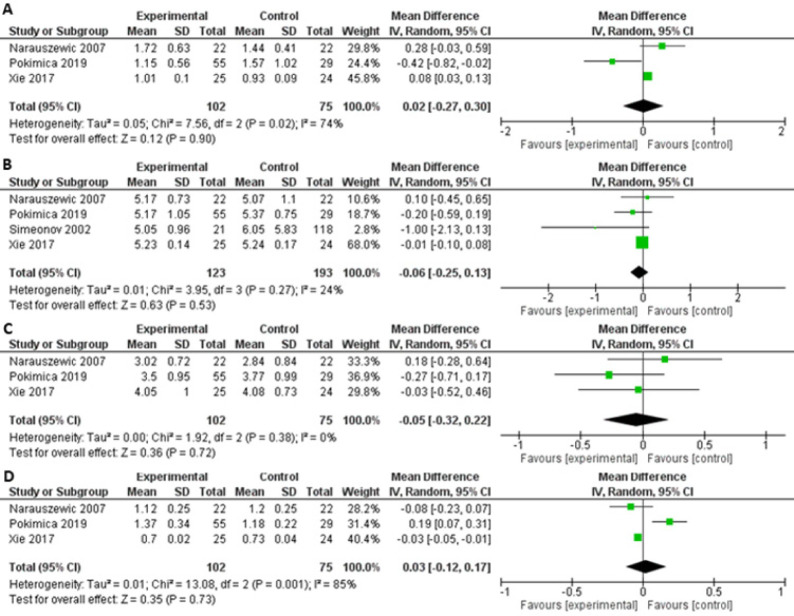
Forrest plot of post-treatment blood lipid concentration in mmol/l (mean difference ± SD) between the intervention and the control groups. A triglyceride, B total cholesterol, C low density lipoprotein and D high density lipoprotein. Abbreviations: CI: Confidence interval.

The quantitative analysis detected no significant decreases in triglycerides, total cholesterol, LDL cholesterol, or HDL cholesterol after consumption of Aronia compared with placebo as Figure 5 illustrates. In 3 RCTs, Aronia extract corresponding to 255 mg to 500 mg polyphenols/day did not lower TG concentration in former smokers, patients with coronary artery disease, or individuals with hypertension or hypercholesterolaemia after supplementation for 4 to 12 weeks [86,90,97,99]. A controlled study which was performed in persons with T2D that daily received 200 ml Aronia juice with unknown polyphenol content for 3 months observed a reduction in total cholesterol [104]. In contrast, 3 RCTs did not detect any effect on total cholesterol after administration of 255 mg to 500 mg polyphenols per day for 4 to 12 weeks [90,97,99]. LDL cholesterol levels were reduced in former smokers after administration of 500 mg polyphenols for 12 weeks [90]. However, in individuals with obesity and coronary artery disease supplementation with 255 mg to 500 mg polyphenols daily for shorter periods did not elicit a lowering of LDL cholesterol [90,99]. None of the studies observed any decreases in HDL cholesterol [90,97,99].

**Figure 6.**
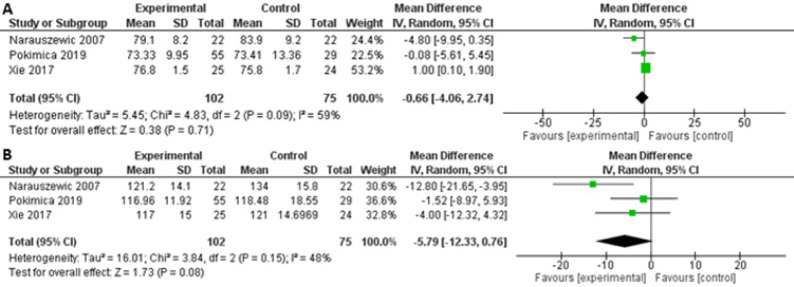
Forrest plot of post-treatment blood pressure in mmHg (mean difference ± SD) between the intervention and the control groups. A diastolic blood pressure and B systolic blood pressure. Abbreviations: CI: Confidence interval.

The quantitative analysis did not detect any overall effect on blood pressure after supplementation with Aronia compared to placebo as Figure 6 illustrates. Two studies with a duration of 4 and 12 weeks, respectively, did not find a positive blood pressure outcome in individuals with MS or former smokers even with administration of 300 mg to 2000 mg polyphenols/ day [90,97]. However, Naruszewich et al. observed decreases in DBP and SBP in patients with coronary artery disease after supplementation with only 285 mg polyphenols/day for 6 weeks [99].

## 4 Discussion

Only 3 studies have examined the effect of Aronia on cardiovascular diseases directly, e.g., T2D and coronary artery disease [91,99,104], and the remaining studies included in this review have investigated the effect of Aronia in participants at increased risk of cardiometabolic diseases [56,68,86,88,90-92,95-100,103,104]. Only 4 studies had a design that made them suitable for the quantitative analysis, which are too few studies to draw firm conclusions. Uncontrolled quasi-experimental studies with a “pre-post” setting with no randomization or blinding have been applied more commonly, thereby increasing the risk of bias. In general, the studies are heterogeneous in design, duration, participants’ risk of cardiovascular disease, and Aronia dosing, which is a limitation in the comparison. Furthermore, Aronia is tested in 2 different formulations, juice and extract, which have different macronutrient composition. Unfortunately, the studies do not provide the exact composition. However, Aronia extracts possibly have a higher content of dietary fibers than juice [11]. As increased intake of dietary fibers may protect against cardiovascular diseases [105], the fibers in Aronia extracts could bias the results if appropriate placebo is not applied. Conversely, fruit juice contains rather high amounts of sugar that quickly raise the blood sugar, which is undesirable in persons with impaired glucose tolerance [106]. However, the included studies do not point towards a blood sugar raising effect of Aronia juice administered to persons with T2D; it even acutely decreased the blood sugar [104].

Regarding the applied doses, Aronia extracts provide polyphenols in concentrated form, which means that participants only had to consume 3 capsules of Aronia extract each day, while Aronia juice was administered in larger amounts of 150 m to 250 ml a day. However, both amounts seem applicable in daily life. It also should be underscored that there is no information about the storage of Aronia juice or extract including recommendations to trial subjects on how to store it. As previously mentioned, polyphenols in Aronia, especially anthocyanins, are known to degrade even at room temperature. Light exposure also increases the degradation rate [44]. If the test products have not been stored in a dark and cold environment, the participants might have received a much lower dose than stated.

Regarding weight loss, overweight individuals do not seem to benefit from supplementation with Aronia for shorter periods than 2 months. The long-term efficacy of the supplementation is unknown, but obese persons may benefit from long-term supplementation with Aronia extract in doses of ≥ 500 mg polyphenols/day [87]. It is also uncertain how Aronia influences blood glucose in T2D. In persons with T2D with high blood glucose at baseline, Aronia juice reduced fasting blood glucose remarkably [91,104], whereas the reduction was limited in patients with lower fasting glucose at baseline [56,68,86,87,95,99]. However, taken together the quantitative analysis indicates that the use of Aronia exerts a lowering of the blood sugar. Thus, Aronia may be beneficial in persons with more severe or poorly controlled T2D, but no conclusions can be drawn due to the low number of studies. In addition, one RCT examining supplementation with a mixture of Aronia, red ginseng, shitake mushroom, and nattokinases in 40 prediabetic individuals found that this mixture compared to placebo had no impact on glucose levels during an oral glucose tolerance test but improved fasting insulin and HOMA-IR index [107]. However, this study does not clarify the impact of Aronia per se, and therefore, is not included in the analysis. On the positive side, Aronia can be considered safe as it did not induce hypoglycaemia [56,68,86,87,99,91,95,104]. However, the overall reduction in blood glucose that was found in the quantitative analysis was too small to have a clinical relevance. It can be anticipated that a higher dosing of Aronia or longer supplementation duration may reveal a positive effect. The impact on insulin sensitivity remains to be determined.

Whereas only one study has investigated the impact of Aronia in persons with established cardiovascular disease [99], several studies have been conducted in persons with one or more cardiovascular risk factors [56,68,86,90-92,95-98,100,104]. A commonality in the studies was that they were of relatively short duration – 4-12 weeks. The participants received either juice or extract, but unfortunately, the polyphenolic content varied substantially between the studies, spanning from around 300 mg to 2000 mg polyphenols administered per day, which hampers the comparisons.

Dyslipidaemia is often present in individuals with cardiovascular diseases, and T2D and constitutes a significant risk for cardiac events [108]. It is questionable whether Aronia supplementation has any beneficial effect on dyslipidaemia. Results on TG [56,68,86,87,90-92,95-97,99] and total cholesterol [56,68,86-92,95-97,99] are conflicting, but baseline values in most studies were low and higher baseline values may reveal a different result. In fact, TG concentration was below 2.2 mmol/l at baseline in all studies except one of the studies that observed a decrease [87]. Genetic interindividual variability also may contribute to the conflicting results [109]. The effect of Aronia also has been evaluated in a meta-analysis that combined both healthy persons and individuals at increased cardiometabolic risk. In opposition to our results, investigators observed a significant reduction in total cholesterol after supplementation with Aronia [110]. However, the results should be interpreted cautiously due to substantial heterogeneity [110]. Even though the quantitative analysis did not find any effect of Aronia on LDL cholesterol, the results from the quasi-designed studies suggest that supplementation with Aronia for 6 weeks to 3 months might decrease LDL cholesterol [87-89,90,91,95,96], while supplementation for shorter periods has no effect [56,68,97,99]. If more long-term studies could have been included in the quantitative analysis, the outcome might have been different. These findings are corroborated by a meta-analysis by Rahmani et al. that has been made on 2 out of 4 of the studies included in our quantitative analysis [109]. It should be noted that in the analysis, baseline LDL cholesterol values were above recommended values in all studies except one. Regarding HDL cholesterol, our results clearly indicate that Aronia has no effect when given in the applied doses for shorter periods of time [56,86-92,95,97,99]. To the contrary, the aforementioned meta-analysis by Rahmani et al. found a small, albeit statistically significant increase in HDL cholesterol [109]. Also, the composition of fatty acid in the blood stream affects the risk of a cardiovascular event, and a high n-3 PUFA concentration is thought to be protective [111]. Aronia may be able to increase especially n-3 PUFA [68,97], but more studies are needed to draw any conclusion. Also, regarding blood pressure, the results are discrepant [56,68,86-88,90-92,95,99] and our quantitative analysis revealed no effect of Aronia, whereas the meta-analysis by Rahmani et al. found DBP increased by 2.55 mmHg but no change observed for SBP [109]. In this context, it is puzzling that a study found a reduction in ACE activity after 2 months supplementation with Aronia, which normally induces a lowering of the blood pressure [88]. On the other hand, the meta-analysis by Hawkins et al. observed no effect on DBP, but a significant effect on SBP [110].

Unhealthy lifestyle, e.g., obesity, smoking, and alcohol consumption increase concentrations of inflammatory markers, of which especially CRP, TNF-α and IL-6 may contribute to the development of insulin resistance and cardiometabolic diseases [112]. Low-grade inflammation is also present in cardiometabolic diseases, and when C-reactive protein (CRP) values exceed 2 mg/l there is a positive correlation between CRP and cardiovascular risk [113,114]. Rahmani et al. did not detect any effect of Aronia supplementation on CRP, IL-1, or TNF-α either [109]. If Aronia could decrease the levels of these markers in individuals with cardiovascular risk, it may prevent or delay disease onset; however, the influence of Aronia on inflammatory markers is not yet clear.

Oxidation of LDL has a detrimental impact on human health, but whether its formation is simply a pathological process or triggered by oxidative stress remain uncertain [115]. The formation of oxidized LDL cholesterol (oxLDL) is a key element in the establishment of arteriosclerotic plaques and a major risk factor for cardiovascular events. In fact, the main goal of lipid lowering treatment is to prevent oxidation of LDL cholesterol [116]. For patients with previous cardiovascular events the recommended LDL cholesterol levels are lower than what can be obtained by a healthy diet alone which makes treatment with statin or other cholesterol lowering drugs inevitable.

However, statin treatment may result in adverse effects, which makes it desirable to discover alternative, effective treatments that could lower oxLDL without inducing side effects [117]. oxLDL decreased in patients with coronary artery disease treated with statins after Aronia supplementation [99], while Aronia supplementation had no effect in individuals at increased cardiovascular risk [97]. More studies are needed to assess whether Aronia could replace statins or reduce the necessary dose. Furthermore, circulating fibrinogen is believed to be correlated to an increase in the risk of cardiovascular diseases [102], but also in this case, further studies need to be done.

Knowledge regarding the uptake and metabolism of the Aronia polyphenols is limited. Some research studies indicate a poor intestinal absorption, which could account for the limited and divergent health benefits found in the literature. Denev et al. reviewed the studies on the absorption of the anthocyanins in Aronia, and even after consumption of a rather large amount of Aronia, only a small fraction of the anthocyanins can be found in the blood, typically in metabolized forms of which the health benefits are unknown [118]. Conversely, Chank et al. investigated the metabolism of cyanidin-3-glucoside as a purified compound in men [119]. After consumption, 24 metabolites were identified in the blood in 46-fold higher concentration than cyanidin-3-glucoside, especially conjugates of protocatechuic acid and hippuric acid, which were abundant. The polymerized procyanidins are most likely not absorbed but exert their effect locally in the colon [118]. Aronia consumption also could exert a positive health effect by modifying the gut microbiota as observed by Istas et al., who reported an increase in Anaerostipes and Bacteroides in healthy men [120]. Interestingly, Bacteroides are reduced in some patients with cardiovascular disease [121] and an increase in Bacteroides might have a positive health impact. Fermentation might improve Aronia’s bioactivity by transforming the polyphenols into other metabolites [122-124].

### 4.1 Limitations

The risk of bias assessment clearly indicates that the overall quality of the studies included in the quantitative analysis is low. Of the studies included in this analysis, only one [90] reports on selection bias and detection bias, while the remaining studies either do not explain the method [97,99] or have a high risk of bias due to lack of randomization and blinding [104]. Unfortunately, Simeonov et al. only have described few details in their study; however, there seems to be some major issues related to their method, e.g., they have not used placebo and it is unclear how they have collected data from the control group [104]. The risk of performance bias in the remaining studies was low, and they all reported using placebo [90,97,99]. Two studies are not registered at clinicaltrials.gov [99,104]. Pokimica et al. have registered their study but performed analyses that were not described at clinicaltrials.gov [97]. Therefore, there is a considerable risk of selective reporting, which increases the possibility that positive outcomes are found by chance.

Unfortunately, Narauszewicz et al. did not use a power calculation, which raises the possibility that the studies are underpowered and unable to detect an effect [99]. Similarly, it is problematic that Xie et al. have significant differences in baseline values, e.g., the control group had been smoking for an average of 15 years while the intervention group members had been smoking for 9 years [90]. Even though the difference is non-significant, the control group is also 5 years older than the control group which could explain the difference in the years of smoking. These differences potentially could mask an effect and indicates problems with their block randomization.

One also should note that age is an important moderator of risk of cardiac events, and the risk is substantially increased after the age of 60 [125]. In relation to the studies included in the quantitative analysis, the average age was below 60 in 3 of the studies [90,97,104], and above 60 in only one study where the participants had a mean age of 66 years [90]. In addition, the low number of studies in the quantitative analysis made evaluations of publication bias and implementation of sensitivity analyses unfeasible.

### 4.2 Conclusion

In conclusion, only few studies have examined Aronia’s impact on cardiometabolic risk factors and diseases. Studies focusing on participants with cardiovascular diseases rather than with increased cardiovascular risk are almost lacking, and the same goes for trials with persons with diabetes where no data are available on the effects of Aronia on insulin secretion and sensitivity. In persons at even higher CVD risk, more pronounced effects may be present. Even though most results are inconsistent, evidence points towards beneficial effects of Aronia on blood glucose levels, but further high-quality RCT studies are needed to allow a clear conclusion. The specific mechanisms of action of polyphenols on cardiometabolic risk factors are not clear. Future studies should be carried out as high quality RCTs performed as long-term studies in patients with cardiometabolic diseases rather than risk factors.

## Abbreviations

FBG: fasting blood glucose HbA1c haemoglobin A1c

TG: triglycerides

TC: total cholesterol

LDL: low density lipoprotein cholesterol

HDL: high density lipoprotein cholesterol

PUFA: polyunsaturated fatty acid

DBP: diastolic blood pressure

SBP: systolic blood pressure

CRP: C-reactive protein

IL: interleukin

TNF-α: tumor necrosis factor-α

MCP-1: monocyte chemoattractant protein-1

oxLDL: oxidized low density lipoprotein

FGN: fibrinogen

ACE: angiotensin-converting enzyme
